# Damaged DNA Binding Protein 2 in Reactive Oxygen Species (ROS) Regulation and Premature Senescence

**DOI:** 10.3390/ijms130911012

**Published:** 2012-09-05

**Authors:** Nilotpal Roy, Srilata Bagchi, Pradip Raychaudhuri

**Affiliations:** 1 Department of Biochemistry and Molecular Genetics (M/C 669), University of Illinois at Chicago, 900 S. Ashland Ave, Chicago, IL 60607, USA; E-Mail: nroy4@uic.edu; 2 Center of Molecular Biology of Oral Diseases (M/C 860), College of Dentistry, Cancer Center, University of Illinois at Chicago, 801 S. Paulina Ave, Chicago, IL 60612, USA; E-Mail: sbagchi@uic.edu

**Keywords:** DDB2, senescence, reactive oxygen species, apoptosis, NER

## Abstract

Premature senescence induced by DNA damage or oncogene is a critical mechanism of tumor suppression. Reactive oxygen species (ROS) have been implicated in the induction of premature senescence response. Several pathological disorders such as cancer, aging and age related neurological abnormalities have been linked to ROS deregulation. Here, we discuss how Damaged DNA binding Protein-2 (DDB2), a nucleotide excision repair protein, plays an important role in ROS regulation by epigenetically repressing the antioxidant genes MnSOD and Catalase. We further revisit a model in which DDB2 plays an instrumental role in DNA damage induced ROS accumulation, ROS induced premature senescence and inhibition of skin tumorigenesis.

## 1. Introduction

Xeroderma Pigmentosum is a rare autosomal hereditary disorder characterized by hypersensitivity to sunlight and predisposition to skin tumor development upon exposure to Ultraviolet light (UV) [1]. The UV rays in sunlight causes variety of DNA damage lesions such as cyclobutane pyrimidine dimers (CPD) and 6-4 photoproducts (6-4 PPs). Cells constantly repair these damages to prevent accumulation of unwanted mutations. Nucleotide excision repair (NER) is particularly important for repair of the aforementioned UV induced DNA damage lesions [2]. NER is a multi-step process requiring involvement of several proteins. Xeroderma Pigmentosum is a genetic defect caused by mutations in the NER enzymes genes, leading to a deficiency in repair and high frequency skin carcinoma development upon UV exposure [3,4]. XP consists of eight complementation groups (XPA, XPB, XPC, XPD, XPE, XPF, XPG and XPV). XPA through XPG encode proteins directly involved in NER. Unlike these Xeroderma Pigmentosum variants, XP-V cells execute normal NER but are defective in the replication of UV damaged DNA. Later studies revealed DNA polymerase eta as the XPV gene product [5].

The XPE gene encodes Damaged DNA binding protein 2 (DDB2), smaller subunit of the damaged DNA binding protein DDB. DDB is a heterodimeric protein complex with a very high affinity to CPDs and 6-4 PPs. DDB recognizes UV damaged DNA as well as DNA damage caused by Cisplatin, abasic sites and single stranded DNA [1]. The DDB2 locus is mapped to 11p12–p11, whereas DDB1 locus is mapped to 11q12–q13. DDB1 gene is strongly conserved among the eukaryotes. Homologs of DDB1 are found in the plant *Arabidopsis thaliana*, the slime mold *Dictyostelium discoideum*, the fission yeast *Schizosaccharomyces pombe*, fruit fly *Drosophila melanogaster* and the nematode *Caenorhabditis elegans*. However, homologs of human DDB2 are found only in higher mammals. DDB2 amino acid sequences between human and mice are only 73% identical. DDB2 is expressed ubiquitously in human tissue with higher expression in testes, liver, kidney, corneal endothelium and lower expression in brain, skin, lung, muscle and heart. There are several isoforms of DDB2: The D1 isoform is the most abundant variant.

Cells from individuals suffering from XPE exhibit a deficient NER activity [6,7]. The NER defect can be restored in XPE cells by microinjection of purified DDB complex demonstrating that DDB2 activity is necessary to carry out the NER process [8]. To characterize the role of DDB2 in NER, several labs generated mice lacking DDB2. Consistent with the phenotype of XP patients, DDB2−/− mice are predisposed to UV induced skin carcinoma formation [9–11]. It is noteworthy that, of all the XP groups, XPE subgroup patients exhibit mildest phenotype with regard to sun sensitivity [1]. Enhanced expression of DDB2 in mice reduced UV induced skin carcinogenesis by both delaying the onset of tumor development and by attenuating the number of tumor occurrence in each mouse [11]. Interestingly, the DDB2−/− mice also develop spontaneous malignant tumor at a very high frequency. Tumors are of wide spectrum with frequent incidence of hematopoietic neoplasms and lymphomas [10,12].

DDB2, along with DDB1, associates with Cul4a, an E3 ubiquitin ligase. Cul4 utilizes WD40-like repeat containing proteins as an adapter molecule. DDB2, also a WD40 repeat containing protein, acts as a substrate receptor molecule for the Cul4a E3 ligase complex, in which DDB1 serves as the linker protein [13]. Interestingly, XPE mutants are unable to bind to Cul4a suggesting role of Cul4a-DDB2 complex in NER [14]. The well-characterized targets of Cul4a-DDB1-DDB2 complex are XPC, p21 and DDB2 [15–17], which are all involved in the repair pathway further underscoring the importance of the protein ubiquitination activity of DDB2.

## 2. Regulation of DDB2

DDB2 is a p53-regulated gene in human [18]. Following UV irradiation, p53 transcriptionally activates DDB2 expression. Further studies revealed a p53 response element in the promoter region of DDB2 [19]. Interestingly, DDB2 was reported also to regulate p53 expression directly [20]. Hence, a positive feedback loop between DDB2 and p53 was suggested, where p53 activates DDB2 expression and DDB2 regulates activation of p53. However, in murine cells there is no p53 response element in the DDB2 promoter region even though mouse DDB2 gene shares significant sequence identity with the human gene [19]. Accordingly, p53 fails to transactivate DDB2 expression following UV irradiation in murine cells.

p38MAPK, a family of serine/threonine protein kinase has been shown to regulate DDB2 expression [21]. p38MAPK has been suggested to facilitate NER following UV irradiation through its phosphorylation of Histone H3 at serine 10 and subsequent alteration of chromatin condensation. This study also indicated that p38MAPK is involved in UV-induced serine phosphorylation of DDB2. Phosphorylation of DDB2 leads to its ubiquitin mediated proteasomal degradation. Degradation of DDB2 results subsequent recruitment of critical pre-incision NER factor XPC and TFIIH. Interestingly, an earlier study reported that c-Abl tyrosine kinase phosphorylates DDB2 and inhibits its activity [22]. However, IR, but not UV augments the tyrosine kinase activity of c-Abl.

Analysis of transcriptional regulatory region of DDB2 revealed core promoter region associated with cell cycle regulating/regulated genes: no TATA box, a G/C rich region, a NF-1 element and multiple Sp1 elements [23]. Furthermore, an active E2F element was also identified on the DDB2 promoter. DDB2 is a cell cycle regulated gene. It is present at a low level in growth arrested primary human fibroblasts. Following release, DDB2 level peaks at G1/S boundary [15]. Part of the cell cycle regulation of DDB2 level involves post-transcriptional mechanism. Further studies revealed that DDB2 is a specific target of Cul4A E3 ligase, which induces proteolysis of DDB2 through the ubiquitin-proteasome pathway [15]. Degradation of DDB2 within hours following UV irradiation could also be explained by Cul4A mediated degradation of DDB2 [11]. Interestingly, DDB2 also associates with COP9 signalosome complex, which participates in proteolysis through ubiquitin-proteasome pathway [24,25].

## 3. Role of DDB2 in NER

NER can be categorized into two distinct pathways: global genomic NER (GG-NER) and transcription coupled NER (TC-NER). GG-NER repairs DNA damage in transcribed and untranscribed DNA strands, whereas TC-NER takes place only in the transcribing DNA strands [26,27]. TC-NER is associated with stalled RNA polymerase II that triggers the repair process, whereas GG-NER is initiated on the genome independently of transcription. NER involves three steps: (a) recognition and unwinding of DNA around the lesion site; (b) single strand excision at both sites of the lesion; and (c) DNA repair synthesis to replace the gap [28]. GG-NER differs from TC-NER at the early recognition step as they employ different proteins to recognize the DNA lesions. In TC-NER, stalled RNA polymerase II results recruitment of Cockayne syndrome A (CSA) and Cockayne syndrome B (CSB) to the DNA damage site, which is followed by the repair process [28]. In GG-NER, XPC-HHR23B and DDB1-DDB2 has been implied to recognize the DNA damage site and initiate the repair process [1,29].

DDB2 participates in the GG-NER [30]. However, the precise role DDB2 plays in NER is a point of controversy. Several models have been proposed. Many of the models involve DDB2s role in protein ubiquitination. Initial reports suggested that DDB2 recruits DDB1 to the nucleus and recognizes the UV induced DNA damage lesions [31–33]. Following damage recognition, Cul4a-DDB1-DDB2 complex recruits XPC at the damage site and ubiquitinates XPC [17]. DDB2 itself also gets self-ubiquitinated. Poly-ubiquitination of DDB2 results into the loss of its DNA binding activity followed by proteasomal-mediated degradation. However, XPC ubiquitination potentiates its DNA binding ability. According to this model, DDB2 degradation facilitates XPC binding to the DNA and activates the repair process [17]. Other models suggest that Cul4a-DDB1-DDB2 complex mono-ubiquitinates histones at the DNA damage site modulating the chromatin structure to help the repair factors gain access to the lesion. Histones H2A, H3 and H4 were identified as the target of Cul4a-DDB1-DDB2 complex to facilitate the removal of histones from the nucleosome at the damage site [34,35]. Moreover, H3/H4 ubiquitination has also been found to play an important role in recruiting XPC to the damaged chromatin [35]. On the other hand, DDB2 was reported to be associated with histone acetyl transferase CBP/p300 [36]. DDB2 was also found to be a component of histone acetylating transcriptional co-activator STAGA [37]. Hence, it was suggested that DDB2 participates in NER by chromatin remodeling at the site of DNA damage. In agreement with that, a recent report provided further evidence that DDB2 plays role in chromatin decondensation at UV induced DNA lesion independent of the protein ubiquitination activity [38]. DDB2 was found to attenuate the density of core histones in the chromatin containing UV induced DNA damage lesions. This function of DDB2 is ATP dependent and involves poly(adenosine diphosphate [ADP]-ribose) polymerase 1. While the observations are compelling, no genetic evidence was provided for these models.

Ataxia telangiectasia mutated kinase (ATM) and Ataxia telangiectasia RAD3-related kinase (ATR) gets activated following DNA damage and recruited to the damaged DNA lesion. Activated ATM/ATR phosphorylates downstream effector p53 at residues Ser15 in human/Ser18 in mouse [39,40]. Ser18 phosphorylation in mouse does not augment the stability of p53, but rather renders p53 transcriptionally more active [41]. In low dose UV irradiated cells DDB2 keeps p53 Ser18P level low, but does not regulate the cellular level of total p53 [42]. DDB2 mediated degradation of p53 Ser18P involves the ubiquitin mediated proteasome pathway. DDB2 imports DDB1 from the cytoplasm to the nucleus following low dose UV. The DDB1-DDB2 complex in association with Cul4a results proteolysis of p53 Ser18P [42]. By degrading p53Ser18P, DDB2 regulates the level of p21 Waf1/Cip1, which is a direct transcriptional target of p53 following DNA damage [43]. Moreover, DDB2 regulates p21 Waf1/Cip1 expression at the protein level through its ability to induce proteolysis of p21 Waf1/Cip1 [16]. p21 Waf1/Cip1 has been reported to impede the DNA repair. Thus, by attenuating expression of p21 Waf1/Cip1, DDB2 ensures efficient DNA repair activity. In agreement with that, deletion of p21 Waf1/Cip1 in DDB2−/− background eliminated the repair deficiency of DDB2−/− cells. These observations provide first genetic evidence on role of DDB2 in the global genomic NER pathway through regulation of p21 Waf1/Cip1.

## 4. Role of DDB2 in Apoptosis

DDB2 is an important mediator of apoptosis following DNA damage [16,44,45]. DDB2 mutated XPE cells were found to be deficient in UV mediated apoptosis response due to severely reduced basal and UV induced p53 level [9]. Later studies revealed that DDB2 deficient mouse embryonic fibroblasts or human carcinoma cells are resistant to most DNA damage (IR, chemotherapeutic drugs or E2F1) induced apoptosis [16]. Also, keratinocytes from DDB2−/− mice are resistant to UV induced apoptosis response. Further studies revealed that the apoptosis function of DDB2 is linked to its ability to attenuate expression of p21 Waf1/Cip1. p21 Waf1/Cip1 exhibits anti-apoptotic effect by multiple mechanisms [46]. One mechanism involves p21 Waf1/Cip1 mediated inhibition of *S* phase progression. p21 Waf1/Cip1 may also inhibit apoptosis by interacting with pro-apoptotic molecules pro-caspase 3, caspase 8, ASK1. In DDB2−/− cells there is high-level accumulation of p21 Waf1/Cip1, which inhibits the apoptosis response. In agreement with that, the DDB2−/− p21−/− cells show efficient apoptosis response after DNA damage. DDB2 has also been implicated in augmenting apoptosis response by chemotherapeutic drug Cisplatin through attenuation of anti-apoptic protein Bcl-2 expression [47].

## 5. Role of DDB2 in Premature Senescence and ROS Regulation

High-level p21 Waf1/Cip1 has been implicated with augmented senescence response [48]. However, quite intriguingly, despite high level of p21 Waf1/Cip1, DDB2 deficient cells were found to be impaired in senescence. For example, MEFs (mouse embryonic fibroblasts) isolated from DDB2−/− embryos are resistant to replicative senescence [44]. p19 Arf (p14 Arf in human), a protein encoded by alternative reading frame product of INK4a/ARF locus, is a critical factor for senescence induction [48]. DDB2−/− MEFs were found to be deficient in p19Arf expression at the later passages. Overexpression of p19Arf rescued senescence deficiency phenotype of DDB2−/− MEFs. Also, DDB2 expression, both at protein and RNA level, was found to be positively correlated with the replicative senescence response of the wild type (WT) MEFs, indicating that DDB2 participates in the senescence response in these cells. MEFs exhibit replicative senescence due to oxidative stress resulted from supra-physiological oxygen concentration (20%) in standard tissue culture condition [49]. In agreement with that, when WT MEFs were cultured at 3% oxygen concentration, there was no induction in DDB2 expression and an inhibition of senescence.

Furthermore, exogenous oxidative stress, such as sub-lethal dose of Hydrogen Peroxide, also increases expression of DDB2 [44]. In the absence of DDB2, both human and murine cells were found to be deficient in premature senescence response, such as oxidative stress, oncogene and DNA damaged induced senescence [44]. For example, reduced expression of DDB2 in human primary fibroblast IMR90 inhibited Ras mediated premature senescence response [44]. Also, DDB2 deficient MEFs or human carcinoma cells were refractory to UV, Cisplatin or Aclarubicin induced senescence [44]. These observations demonstrated that DDB2 is an essential mediator of premature senescence response. Further investigation revealed that DDB2 is required for persistent accumulation of ROS, a critical regulator for premature senescence [44]. Senescence function of ROS is attributed to its ability to induce p53 and MAPK family members-P38MAPK, JNK and ERK1/2 and subsequent irreversible cell cycle arrest at G1 phase [50].

In both mouse embryonic fibroblasts and human carcinoma cells, the DDB2 deficiency was found to impair ROS accumulation following DNA damage (UV, Cisplatin or Aclarubicin treatment) [44]. Furthermore, DDB2−/− mice accumulated significantly less ROS in the skin following acute UV damage [51]. High-levels of ROS are lethal oxidants for the cell. Hence, cells employ anti-oxidant defense mechanism to neutralize ROS [52]. ROS are regulated in the cell by plethora of enzymatic and non-enzymatic mechanisms. Superoxide dismutase, Catalase, and Glutathione peroxidase are the major anti-oxidant enzymes. The major non-enzymatic anti-oxidant molecules include Glutathione, Thioredoxin, Vitamin A, Vitamin C, and Vitamin E [52]. Investigation into the mechanism of DDB2 mediated ROS regulation revealed that DDB2 is a transcriptional inhibitor of two important anti-oxidant enzymes, MnSOD and catalase.

Superoxide dismutases catalyze the dismutation reaction of superoxide into oxygen and hydrogen peroxide. There are three major families of superoxide dismutases based upon the metal co-factor: Cu/ZnSOD (co-factor is copper and zinc), MnSOD (co-factor is manganese) and NiSOD (co-factor is Nickel). Eukaryotes use Cu/ZnSOD and MnSOD, whereas prokaryotes use MnSOD or NiSOD. In mammals, the three kinds SOD1, SOD2 and SOD3 differ in their localization. SOD1 is found in the cytoplasm; SOD2 is found in mitochondria and SOD3 is extracellular. SOD1 and SOD3 are Cu/Zn SODs whereas SOD2 is MnSOD. SOD2−/− mice have an extremely short lifespan, as within first ten days of their life they die due to dilated cardiomyopathy, accumulation of lipid in liver and skeletal muscle and metabolic acidosis [53]. Another study reported neurodegeneration of SOD2−/− mice along with above-mentioned abnormalities [54]. Catalase is another enzyme critical for anti-oxidant defense. It catalyzes the decomposition of hydrogen peroxide to water and oxygen. Catalase is present in a cell organelle known as peroxisome. Surprisingly, the Catalase−/− mice develop normally and do not show any overt abnormalities [55].

Previously, DDB2 has been implicated in playing a role in transcription. For example, DDB2 has been shown to play the role of co-activator for E2F1 mediated transcriptional activation [56]. Also, DDB2 is associated with histone modifying enzymes such as CBP/p300 histone acetyl transferase and STAGA complex, a histone acetylating transcriptional co-activator [36,37]. DDB2 binding partner Cul4a-DDB1 has also implicated in transcriptional regulation. For example, Cul4a has been shown to recruit histone methyltransferase MLL1 onto the p16INK4a promoter to induce expression of p16INK4a during oncogenic stress [57]. In fission yeast, *Schizosachhraomyces pombe*, Cul4 was shown to recruit Clr4 on the promoter of genes [58]. Clr4 is a histone methyltransferase that results Histone 3 Lysine 9 tri-methylation (H3K9 Me3), a heterochromatinizing modification on the promoter of genes. Moreover, Rik1, a fission yeast protein related to mammalian DDB1, was found to be crucial for Cul4 mediated recruitment of Clr4 histone methyl transferase. A recent study identified a cognate binding element for DDB2 on the promoter of MnSOD. That study further suggested DDB2 to be a constitutive repressor of the MnSOD gene [59]. However, our results demonstrated that DDB2 binds to the promoter of both catalase and SOD2 genes, even though the former does not have the cognate binding element for DDB2. Cul4a-DDB2 complex recruits histone methyltransferase Suv39h, the mammalian counterpart of Clr4, on the promoter of these two genes, leading to attenuation of their expression. Thus DDB2 acts as an epigenetic regulator of MnSOD and catalase genes. Interestingly, another study identified many WD40 repeat-containing proteins interacting with Cul4-DDB1 to be core component of histone methylating complex that are essential for histone H3 methylation and epigenetic control at K4, K9 or K27 [60]. It appears that DDB2 limits expression of the anti-oxidant genes by modifying the chromatins of MnSOD and Catalase, leading to ROS accumulation following DNA damage. Furthermore, ROS augment expression of DDB, both at the protein and RNA level. The High-level of ROS induces a premature senescence response. Thus DDB2 acts as a central regulator in a positive feedback loop. ROS stimulate DDB2 expression. DDB2 in turn results persistent accumulation of the ROS by downregulation of anti-oxidant genes Catalase/MnSOD, leading to the premature senescence response.

## 6. Physiological Significance of DDB2 Mediated ROS Regulation

**Figure 1:**
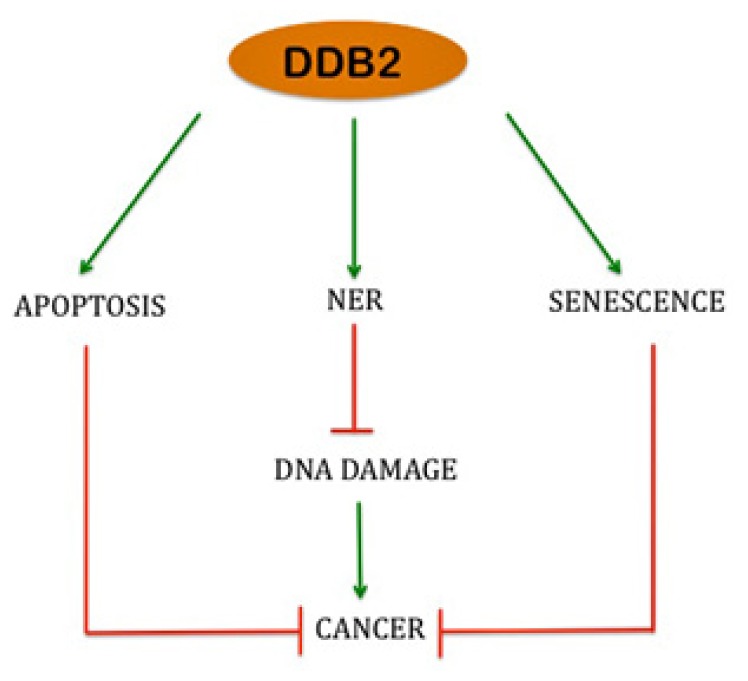
Schematic diagram depicting how DDB2 acts as a tumor suppressor by regulation of nucleotide excision repair, apoptosis and senescence.

The tumor suppression function of DDB2 has been attributed to its in NER and apoptosis pathway [45]. However, recent studies from our group suggests that DDB2 mediated premature senescence response also serves an important part in its role as an inhibitor of UV induced skin carcinoma formation. DDB2−/− mice are deficient in apoptosis as well as repair of UV induced lesions following UV irradiation. *In vitro* studies suggested that accumulation of p21 Waf1/Cip1 in DDB2 deficiency causes this apoptosis and repair deficiency. Concordantly, DDB2−/− p21−/− mice exhibit efficient repair and apoptosis response following UV irradiation [51]. Surprisingly, in spite of efficient apoptosis and DNA repair function, DDB2−/− p21−/− develop skin tumor more aggressively than either WT or DDB2−/− mice [51]. Further investigation revealed that there was an acute deficiency in premature senescence response in DDB2−/− p21−/− mice following UV irradiation. The deficiency is associated with decrease in ROS accumulation in the skin of these mice. Also, there is increased catalase expression in the skin of the mice explaining the deficiency in ROS accumulation. DDB2−/− p21−/− mice also exhibited higher accumulation of transcriptional activator FoxM1, expression of which is inhibited by p21 Waf1/Cip1 [61]. Therefore, quite intriguingly, in the context of UV induced skin carcinoma progression, DDB2 and p21 Waf1/Cip1 cooperate with each other to inhibit tumorigenesis by induction of premature senescence. Therefore, the senescence induction by maintaining high ROS level is an important pathway through which DDB2 acts as a tumor suppressor (Figure1).

Hepatic stellate cells contribute to fibrotic response in the liver following chronic liver damage. Upon withdrawal of chronic damage, hepatic stellate cells undergo premature senescence or apoptosis and get removed from the system leading to regression of liver fibrosis. Deficiency of hepatic stellate cell senescence keeps these cells functionally active, causing an impaired fibrotic response [62]. For example, p53−/− or p53−/− INK4a/ARF−/− mice are deficient in restricting fibrotic regression following withdrawal of chronic damage owed to their deficient senescence response. Similarly, DDB2−/− mice were found to be deficient in senescence of liver cells following withdrawal of chronic liver damage, suggesting that DDB2 might play a critical role in controlling liver fibrosis [44].

Interestingly, a recent study reported DDB2 to be one of the top ten biomarkers of aging by transcript profiling of whole blood [63]. Authors of this study examined age reflecting shift in transcript balance in the peripheral blood mononuclear cells of individuals regardless of gender between age 23 and 77. After examining two cohorts of 153 healthy individuals, they identified sixteen transcripts that exhibit strong and reproducible age related shift. DDB2 was reported to be one of the transcripts whose expression levels positively correlated with age. Authors proposed that the identified genes play a critical role in senescence associated dynamic shift in the immune activity of aged individuals. However, given the important role DDB2 plays in oxidative stress induced premature senescence response, it is plausible that the oxidative stress associated with age increases DDB2 expression. According to the free radical theory of aging, ROS generated in one’s lifetime results degenerative diseases associated with aging. Therefore, it will be interesting to examine whether increased ROS production causes DDB2 accumulation in the aging individuals. If that is the case, then it is tempting to explore whether DDB2 can be targeted for antioxidant therapy.

Two different studies reported a significant association of a SNP (single nucleotide polymorphism) in the DDB2 gene to age related disorders in individuals. One study found a significant association of a SNP in the promoter region of DDB2 gene with carotid-femoral pulse wave velocity that measures the artery stiffness [64]. Artery stiffness results vascular dysfunction related to atherosclerosis and diabetes in the aging individuals. The other study identified DDB2 locus to be associated with the neurodegenerative disease progressive supranuclear palsy, the second most common form of Parkinsonism [65]. These evidences provoke further studies to explore the role of DDB2 in aging and age related disorders.

## 7. Concluding Remarks

**Figure 2:**
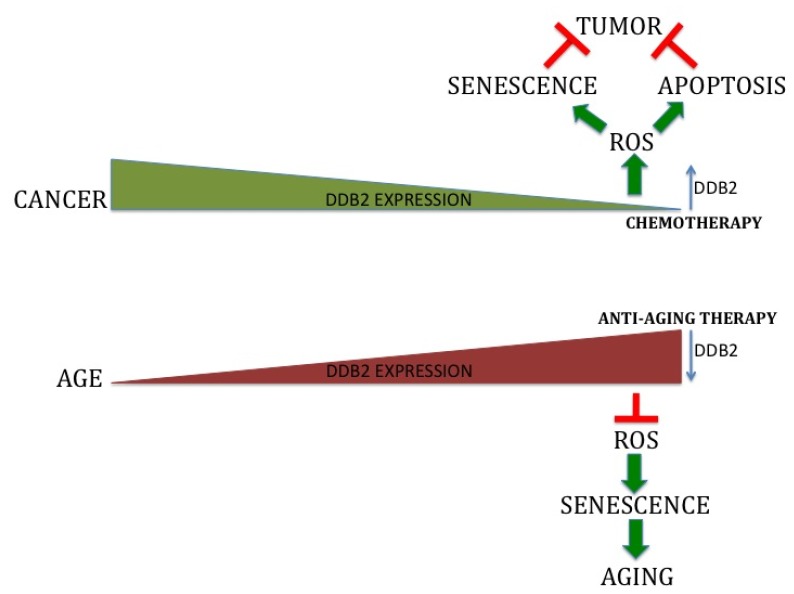
Schematic diagram depicting how DDB2 can be targeted therapeutically for the treatment of cancer and aging. DDB2 expression is reduced during carcinoma progression. DDB2 up-regulation can be therapeutically achieved to induce senescence and apoptosis response to inhibit tumorigenesis. In contrast, DDB2 expression is augmented with aging. Hence, attenuation of DDB2 expression might prove to be beneficial to inhibit aging and age related disorders.

Senescence is often regarded as a classic case of antagonistic pleiotropy [66]. Antagonistic pleiotropy is defined as the production of multiple contrasting phenotypic effects by a single biological process. Generally, some of the effects are beneficial to the organism, whereas the others are detrimental. Senescence is preferred by natural selection to promote fitness and survival advantage in young organisms (*i.e*., suppressing cancer) over its detrimental effect in aged organisms (*i.e*., age related pathophysiologies). In young individuals increased DDB2 expression is beneficial to prevent cancer by induction of senescence and apoptosis. However, at a later stage of life, high-level DDB2 might be detrimental because of increased ROS accumulation and ROS-associated abnormalities (Figure 2). Hence, regulation of DDB2 gene expression and, in turn, DDB2 mediated gene expression might vary in different contexts. Further, DDB2 expression is attenuated in a wide variety of cancers at the RNA level [67]. It has been validated at the protein level as well in basal cell carcinoma patient samples where there is evidence for loss of DDB2 expression compared to the normal tissue [51]. Hence, it is important to recognize how DDB2 expression is lost during carcinoma progression and will be important to determine whether restoration of DDB2 expression helps to prevent tumor development. On the other hand, it is also necessary to examine how DDB2 is stabilized during aging that leads to age related disorders. DDB2 is a p53-induced gene. Therefore, it is a possibility that DDB2 expression is attenuated with the loss of p53 function in cancer. Also, DDB2 transcription start site is TATA-less, G/C rich and consists of Sp1 and NF1 elements [23]. Housekeeping genes and cell cycle regulated genes are frequently associated with aforementioned elements. These observations clearly indicate that DDB2 is under tight gene expression control and there might be several other factors that regulate its expression. As DDB2 promoter is G/C rich, it will be important to examine whether there is an increased hypermethylation of the CpG islands in the promoter of DDB2 with the carcinoma progression. For several genes, such as p16INK4a, promoter hypermethylation mediated repression has been evidenced in cancer cells [68]. Restoration of DDB2 expression will induce ROS accumulation that would inhibit tumor progression through senescence or apoptosis. Can any pharmacological agent augment re-expression of DDB2? The ROS-mediated expression of DDB2 can be exploited to elevate DDB2 expression. There are several pharmacological agents that are currently being explored to promote ROS generation in cancer cells as a means to kill the cells. These drugs act on the transformed cells selectively as they exhibit elevated ROS generation associated with active metabolism and oncogenic stimulation. Some of these agents directly induce ROS generation such as Motexafin Gadolinium, which is a pro-oxidant catalyst and induces intracellular superoxide generation. Agents that inhibit elimination of ROS have been more successful in the treatment of patients. These drugs target different anti-oxidant pathways. Phenylethyl Isothiocyanate (PEITC) and Imexon cause depletion of GSH [69]. Also, the SOD inhibitors, such as 2-methoxyestradiol and Tetrathiomolybdate, and catalase inhibitors, such as Mangafodipir increase ROS. Many of these pharmacological agents are already in phase II/III clinical trial and proved to be effective in the treatment of certain types of cancer [69]. PEITC, a natural compound found in cruciferous vegetables, has been reported to be effective against wide variety of cancers [69,70]. PEITC has been reported to inhibit ovarian and prostate tumor progression [71]. It is presently in clinical trial for the treatment of lung cancer and lymphoproliferative disorders. Hence, being a natural compound with less toxicity, PEITC seems like an attractive agent for induction of DDB2 in cancer patients. PEITC induced DDB2 expression will keep the ROS level high by down regulation of anti-oxidant genes MnSOD and catalase and trigger apoptosis/senescence of transformed cells.

Also, it will be interesting to explore whether DDB2 attenuation have any effect on aging or age related disorders. Certainly, high level of ROS is detrimental for age related pathophysiology. Hence, it will be important to examine whether reduction in ROS level with reduced expression of DDB2 have beneficial effect at later stages of life. In conclusion, the discoveries on DDB2’s transcriptional function have revealed several interesting possibilities that can be explored in designing novel therapeutics for the treatment of cancer and aging.
